# The Utility of Supertype Clustering in Prediction for Class II MHC-Peptide Binding

**DOI:** 10.3390/molecules23113034

**Published:** 2018-11-20

**Authors:** Wen-Jun Shen, Xun Zhang, Shaohong Zhang, Cheng Liu, Wenjuan Cui

**Affiliations:** 1 Department of Bioinformatics, Shantou University Medical College, Shantou 515000, China; 17xzhang6@stu.edu.cn (X.Z.); chengliu10@gmail.com (C.L.); 2 Department of Computer Science, Guangzhou University, Guangzhou 510000, China; zimzsh@gmail.com; 3 Computer Network Information Center, Chinese Academy of Sciences, Beijing 100190, China; wenjuancui@cnic.cn

**Keywords:** class II MHC, MHC-peptide binding, supertype, ensemble learning

## Abstract

Motivation: Extensive efforts have been devoted to understanding the antigenic peptides binding to MHC class I and II molecules since they play a fundamental role in controlling immune responses and due their involvement in vaccination, transplantation, and autoimmunity. The genes coding for the MHC molecules are highly polymorphic, and it is difficult to build computational models for MHC molecules with few know binders. On the other hand, previous studies demonstrated that some MHC molecules share overlapping peptide binding repertoires and attempted to group them into supertypes. Herein, we present a framework of the utility of supertype clustering to gain more information about the data to improve the prediction accuracy of class II MHC-peptide binding. Results: We developed a new method, called superMHC, for class II MHC-peptide binding prediction, including three MHC isotypes of HLA-DR, HLA-DP, and HLA-DQ, by using supertype clustering in conjunction with RLS regression. The supertypes were identified by using a novel repertoire dissimilarity index to quantify the difference in MHC binding specificities. The superMHC method achieves the state-of-the-art performance and is demonstrated to predict binding affinities to a series of MHC molecules with few binders accurately. These results have implications for understanding receptor-ligand interactions involved in MHC-peptide binding.

## 1. Introduction

Major histocompatibility complex (MHC) molecules, called human leukocyte antigen (HLA) in humans, act as cell surface vessels, which hold antigen fragments within their binding groove for recognition by T cells. There are two main classes of MHC molecules: class I and II, which differ in terms of which cells express them, in the source of the antigens they bind to, and in terms of which T cells they present antigen pieces to [1,2]. Class I MHC molecules exist on the surface of nearly all nucleated cells and specialize in displaying antigenic peptides that originate from the cytosol. CD8${}^{+}$ T cells recognize the complex of the class I MHC molecules plus antigenic peptides and kill cells expressing such intracellular antigens. On the other hand, class II MHC molecules are predominantly expressed on antigen-presenting cells (B cells, macrophages, and dendritic cells) and specialize in displaying antigen fragments that originate from extracellular spaces. CD4${}^{+}$ T cells recognize those foreign peptides in complex with class II MHC molecules and then produce a large number of cytokines to activate various cells toward destroying extracellular invaders [3].

Class II MHC molecules are composed of two different chains: α (33 kDa) and β (28 kDa), each of which consists of two external domains: α1 and α2 domains in the α chain and β1 and β2 domains in the β chain. The peptide-binding cleft of class II MHC molecule is formed by the α1 and β1 domains. X-ray crystallographic study reveals that the binding clefts of class I and II MHC molecules are different at both ends: MHC class I is closed at both ends, while MHC class II is open at both ends. The reason is class I MHC molecules have conserved residues that bind to the terminal residues of antigenic peptides, while these kinds of conserved residues do not exist in the class II MHC molecules. Class II MHC molecules can accommodate longer peptides than class I MHC molecules, which results in increased difficulty in performing accurate prediction for class II MHC-peptide binding. In humans, the α and β chains of class II MHC molecules are encoded by the DP, DQ, and DR loci. Each MHC locus encodes numerous allele variants [2]. By May 2018, the IPD-IMGT/HLA database [4] release listed 2450 DRB (the abbreviation for the gene encoding the class II HLA-DR β-chain) alleles, 1193 DQB (HLA-DQ β-chain) alleles, and 974 DPB (HLA-DP β-chain) alleles. Such high levels of polymorphism presumably enhance the probability of whole species survival via a wide range of infectious diseases, but cause difficulty for the designation of high population coverage vaccines. The supertype identification problem essentially is to identify peptides that can bind to a set of MHC molecules, aiming at reducing the total number of epitopes for multi-epitope vaccines’ design without compromising population coverage [5,6]. The first class I HLA supertypes were proposed by [5], who defined nine supertypes based on clustering the structural motifs derived from experimentally-determined binding data. Some MHC molecules share significant peptide-binding repertoires despite apparent motif differences [7]; thus, some methods have been developed to classify HLA molecules into supertypes by considering the peptide-binding repertoires [8,9,10,11]. However, most of these works studied peptide-binding specificities by using peptide-binding repertoires predicted by computational approaches, such as the position-specific scoring matrix (PSSM) [8] or neural networks [10,11]. The work described in [9] used actual peptide-binding measurements, but the binding repertoire was less than 90 peptides for each molecule.

The interactions of T cells with MHC-peptide complexes play a vital role for the T or B lymphocytes to proliferate and differentiate into effector cells or memory cells [2]. Previous studies have clarified that the MHC-peptide binding strength has a strong correlation with peptide immunogenicity [12,13]. Many different approaches for the prediction of class II MHC-peptide binding have been developed, including TEPITOPE [14], TEPITOPEpan [15], NetMHCII [16], NetMHCIIpan [17], KernelRLS, and KernelRLSPan [18]; however, a few methods among them [10,11] can make the prediction for HLA-DP and HLA-DQ molecules.

In this study, a large-scale dataset derived from quantitative MHC binding assays was employed to characterize supertypes from the 41 most common class II HLA molecules covering the DR, DP, and DQ loci. To make meaningful comparisons between peptide-binding repertoires, we developed a novel repertoire dissimilarity index (RDI) as a measure of distance between them to quantify the difference in MHC binding specificities. Furthermore, we explored the utility of supertype clustering in prediction for class II MHC-peptide binding, including three class II HLA isotypes of HLA-DR, HLA-DP, and HLA-DQ.

## 2. Results and Discussion

### 2.1. Identification of HLA II Supertypes

In the present study, a large-scale dataset derived from quantitative MHC binding assays was employed to characterize supertypes from 41 class II HLA molecules covering the DR, DP, and DQ loci. This dataset (see Table 1 for more details) contained 96,674 class II MHC-peptide pairs with IC${}_{50}$ measurements, in which 46,113 pairs were associated with good (IC${}_{50}$ ≤50 nM) or intermediate binding (50 nM < IC${}_{50}$ ≤500 nM).

**Figure 1:**
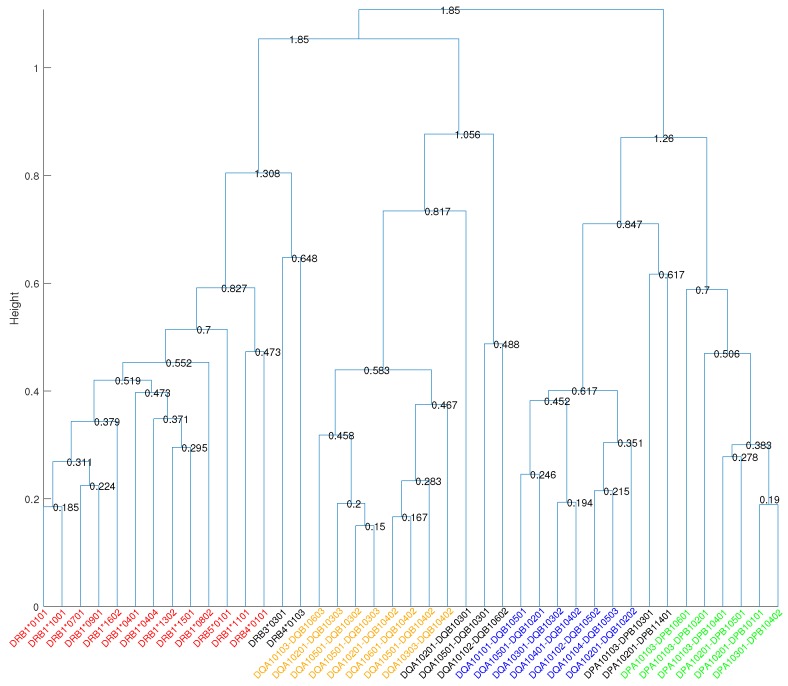
Cluster tree on 41 class II HLA molecules. The height of each horizontal line corresponds to the distance between the two clusters it merges. The numbers given in the figure are the diameters of the corresponding unions of clusters. Four main clusters, main DR, main DQ1, main DQ2, and main DP, are highlighted in red, orange, blue, and green, respectively.

**Figure 2:**
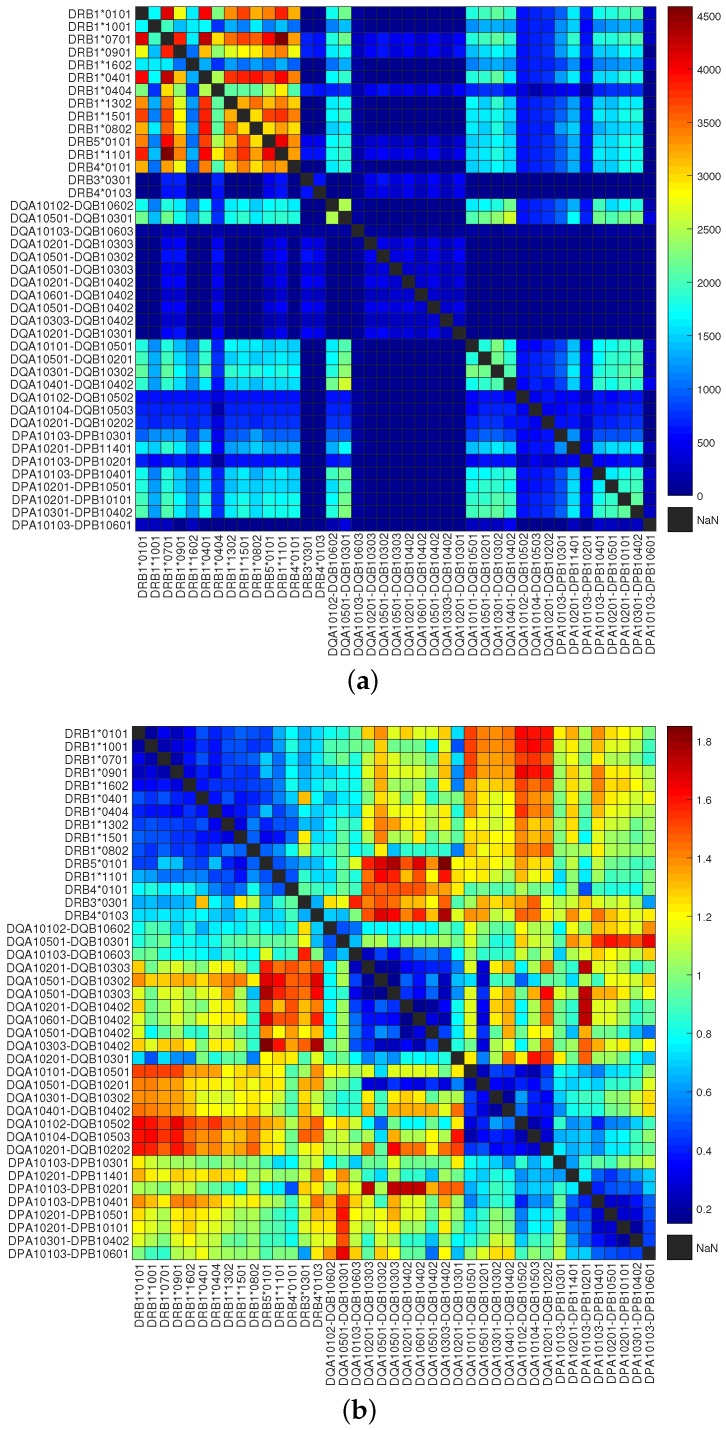
Comparison of the number of peptide binders with repertoire dissimilarity index (RDI) values of class II HLA molecules. (**a**) Heatmap of the number of peptide binders shared by HLA molecules; (**b**) heatmap of the RDI values between HLA molecules.

To quantify the overlapping MHC-peptide binding repertoires, we developed the RDI as a measure of distance between repertoires. This metric uses Kendall’s rank correlation coefficient [19] to ascertain the degree of association among a given set of peptide-binding repertoires for two MHC molecules. Using the RDI, we generated a dissimilarity matrix whose entries quantified the difference in MHC binding specificities. Hierarchical clustering [20] of this dissimilarity matrix revealed four main supertypes covering the 34 most common HLA DR, DP, and DQ molecules (Figure 1). The cut-off distance for the formation of clusters was set to 0.7 to exclude negative and weaker correlations. We can see that cutting the cluster tree at this level separates HLA molecules at different loci. As shown in Figure 2, the high variance in different MHC peptide-binding repertoires resulted in orders of magnitude difference in counts of sharing binding peptides (Figure 2a), while the matrix of RDI clearly displayed four blocks on the diagonal, which was in accordance with the four main supertypes (Figure 2b).

We identified two main supertypes for HLA-DQ and one each for HLA-DR and HLA-DP. The HLA molecules within the supertypes are all encoded by either the DR, DP, or DQ locus, and no cross-loci supertypes were found, which is consistent with the earlier findings [9,10,11]. The cluster tree in Figure 1 shows that the main DQ1 supertype was closer to the main DR supertype, while the main DQ2 supertype was closer to the main DP supertype. The average Kendall’s correlation coefficient for molecules between main DQ2 and main DP was 0.169, and it was close to zero or negative for molecules between other main supertypes, indicating very weak positive correlations between distinct main supertypes. Furthermore, we assigned split supertypes to certain HLA molecules in the cluster tree based on the common binding motifs previously described [11]. Table 2 lists the broad supertypes in connection with the split supertypes and the corresponding peptide motifs.

**Figure 3:**
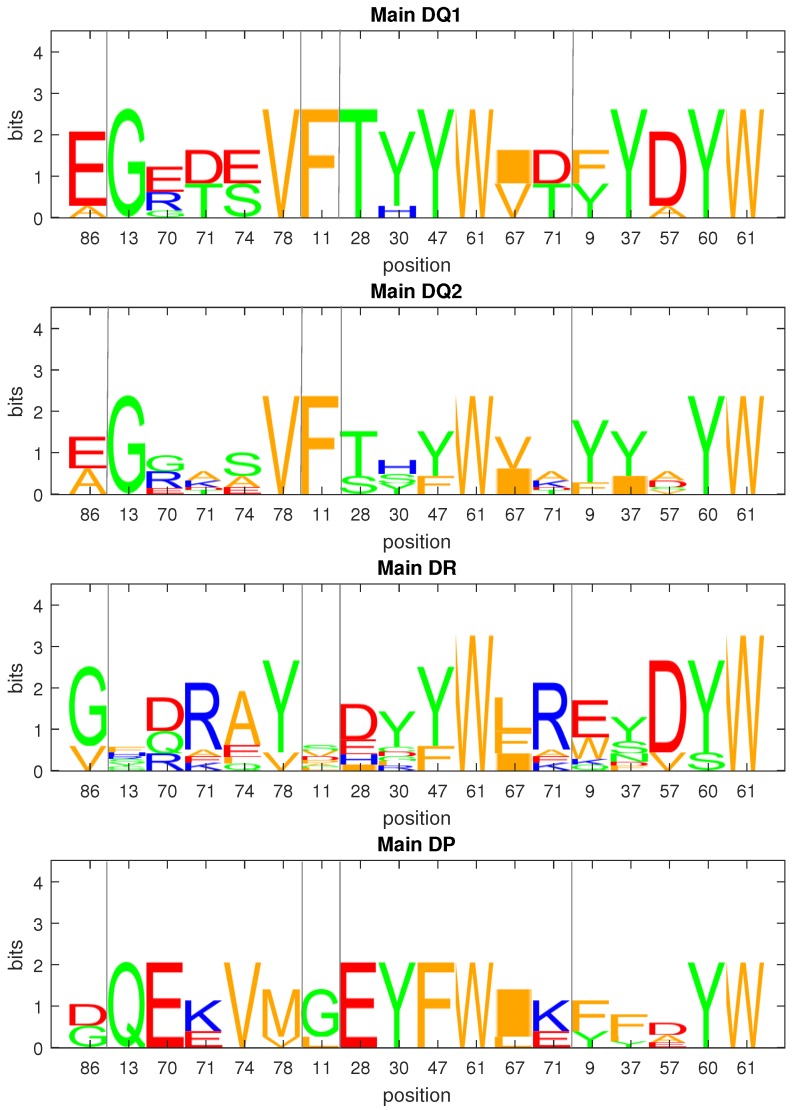
Sequence logos for anchor pocket residues of MHC II β chains. The residues making up anchor pockets 1, 4, 6, 7, and 9 are separated by gray vertical lines. Pocket 1 consists of residue 86. Pocket 4 consists of residues 13, 70, 71, 74, and 78. Pocket 6 consists of residue 11. Pocket 7 consists of residues 28, 30, 47, 61, 67, and 71. Pocket 9 consists of residues 9, 37, 57, 60, and 61. The residues are numbered based on the HLA-DR β chain.

For class II MHC molecules, the β chain is much more polymorphic than the α chain; thus, we compared the sequence motifs of the polymorphic pocket residues on the β chain, as shown in Figure 3. For the HLA-DR β chain, pockets 4, 6, 7, and 9 are responsible for the peptide binding specificity and are mainly formed by the polymorphic residues, as described in the work of Tiziana et al. [14]. Pocket 1 plays an important role in determining the binding core. The HLA-DP and HLA-DQ molecules were demonstrated to have minor variations from HLA-DR in the peptide-binding domain [10]; therefore, we investigated their anchor pockets by using the same residues as the HLA-DR’s. Residue 86 takes part in the formation of pocket 1. From the sequence logos in Figure 3, the β chains of HLA-DR, -DQ, and -DP have Gly/Val${}^{86\beta }$, Glu/Ala${}^{86\beta }$, and Asp/Gly${}^{86\beta }$, respectively. In contrast to Gly/Val${}^{86\beta }$ making up a deep and nonpolar pocket, pocket 1 with Glu${}^{86\beta }$ and Asp${}^{86\beta }$ was more shallow and negatively charged; however, all class II MHC isotypes share a strong preference for large hydrophobic residues in position 1 of the peptide binding core (see Table 2). In contrast, the P4, P6, P7, and P9 motifs are more diverse across different isotypes and are described in the following subsections.

### 2.2. HLA-DR Supertypes

In our analysis, the molecules included in the main DR cluster matched very well to the previously described “main DR supertype” [6,9]. The study in [6] demonstrated that a set of seven HLA-DR molecules (DRB1*0101, DRB1*0401, DRB1*0701, DRB5*0101, DRB1*1501, DRB1*0901, and DRB1*1302) shared overlapping peptide binding repertoires by using a panel of quantitative assays. The main DR supertype described in this study identified a larger set of HLA-DR molecules, characterized by overlapping peptide-binding repertoires, which not only covered all of those seven molecules described earlier, but also identified six additional HLA-DR molecules (DRB1*0404, DRB1*0802, DRB1*1001, DRB1*1602, DRB1*1101 and DRB4*0101). The average Kendall’s correlation coefficient between molecules within the main DR supertype was 0.526. The cluster tree (Figure 1) describing the HLA-DR molecules was characterized by three split supertypes, called DR1, DR52, and DR53, based on common binding motifs predicted in [11]. As shown in Table 2, the DR1 cluster comprising DRB1*0101, DRB1*1001, DRB1*0701, DRB1*0901, DRB1*1602, DRB1*0401, DRB1*0404, and DRB1*1501, can be characterized by a shared preference for hydrophobic residues (F, Y, I, L) in position 1 [9], small and/or aliphatic residues (L, A, I, S, T) in position 4, small residues (A, S, G, P, T) in position 6, and hydrophobic and aliphatic residues (L, V) in position 9 of the 9-mer binding core. The DR52 cluster includes DRB3*0301 and DRB1*1302, while the DR53 cluster includes DRB1*0802, DRB5*0101, DRB1*1101, DRB4*0101, and DRB4*0103. Both DR52 and DR53 clusters showed a strong preference for hydrophobic residues in position 1, as well. The DR52 cluster was characterized by a preference for peptides bearing hydrophilic residues (N, S) in position 4. The DR53 cluster was observed to prefer positively-charged amino acid R in position 2 and the positions near the C-terminal end of the binding core (P5–P9). The anchor positions 1, 4, 6, and 9 at the binding core were identified to govern the binding strength of peptides with class II MHC molecules [3] and were observed to dominate the HLA-DR peptide binding specificities, as well.

### 2.3. HLA-DQ Supertypes

The data presented herein suggest that there are two main supertypes for HLA-DQ molecules, called main DQ1 and main DQ2. The average Kendall’s correlation coefficient between molecules within main DQ1 and main DQ2 was 0.643 and 0.638, respectively, while it was 0.009, near zero, for molecules between main DQ1 and main DQ2. From the sequence logos shown in Figure 3, the residues 70/71/74 composing pocket 4, 30/71 in pocket 7, and 9/57 in pocket 9 play an important role in DQ classification.

The HLA-DQ molecules were grouped into five split supertypes, as summarized in Table 2, which is highly consistent with the groups in the distance tree presented in [11]. All split supertypes shared strong specificities for hydrophobic residues in position 1. The anchor positions of DQ7 cluster were in positions 1, 3, 4, 6, and 9. The peptide motifs of the DQ7 cluster comprising DQA1*0501-DQB1*0301, DQA1*0201-DQB1*0301, DQA1*0102-DQB1*0602, DQA1*0103-DQB1*0603, DQA1*0201-DQB1*0303, DQA1*0501-DQB1*0302, and DQA1*0501-DQB1*0303 were observed to bear small and/or hydrophobic residues in positions 3, 4, 6, and 9. The DQ4 cluster (DQA1*0201-DQB1*0402, DQA1*0303-DQB1*0402, DQA1*0601-DQB1*0402, and DQA1*0501-DQB1*0402) recognized a broad motif characterized by hydrophobic residues (F, A, W) in position 4, small and/or hydrophobic residues in positions 6 and 9, and positively-charged residue R and amino acid A in position 7. The DQ5 cluster included DQA1*0102-DQB10502, DQA1*0104-DQB10503, and DQA1*0101-DQB10501, whose peptide motifs were characterized by a shared preference for aromatic residues in position 4 and acidic amino acid D in positions 6 and 7. The clusters DQ2 and DQ8 preferred acidic residues (D, E) in the positions P4–P9 and P7–P9, respectively.

### 2.4. HLA-DP Supertypes

The cluster tree grouped most HLA-DP molecules into a main HLA-DP supertype that comprises DPA1*0103-DPB1*0401, DPA1*0201-DPB1*0101, DPA1*0201-DPB1*0501, DPA1*0301-DPB1*0402, DPA1*0103-DPB1*0201, and DPA1*0103-DPB1*0601. The average Kendall’s correlation coefficient between molecules within the main DP cluster was 0.590. In our analysis, the HLA-DP molecules were classified into two split supertypes, called DP1 and DP3. The DP1 cluster was characterized by a consensus motif including large hydrophobic residues (F, L, Y, I) in positions 1 and 6 and amino acid L in positions 7 and 9. The DP3 cluster, including DPA1*0103-DPB1*0301, and DPA1*0201-DPB1*1401, also favors large hydrophobic resides in position 1, but strongly prefers positively-charged residues (R, K) in position 2 and small and/or hydrophobic residues in positions 4, 6, and 7.

### 2.5. Class II MHC-Peptide Binding Prediction

**Figure 4:**
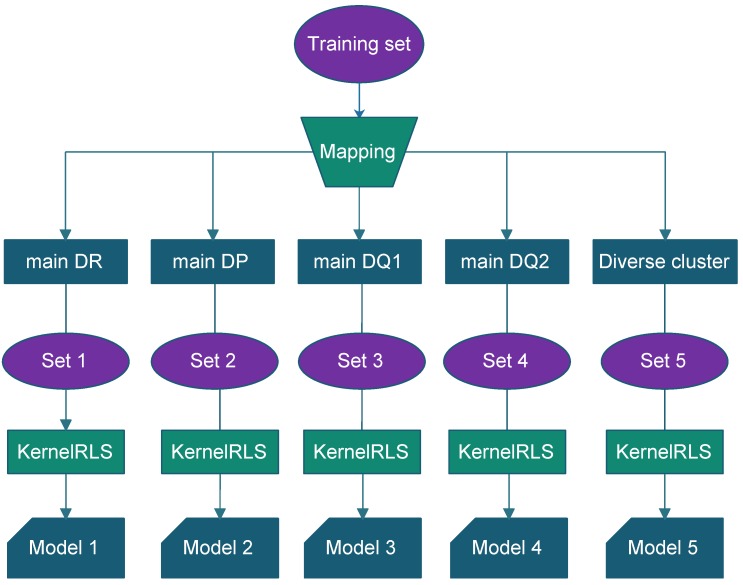
Schematic illustration of generating multiple prediction models.

**Figure 5:**
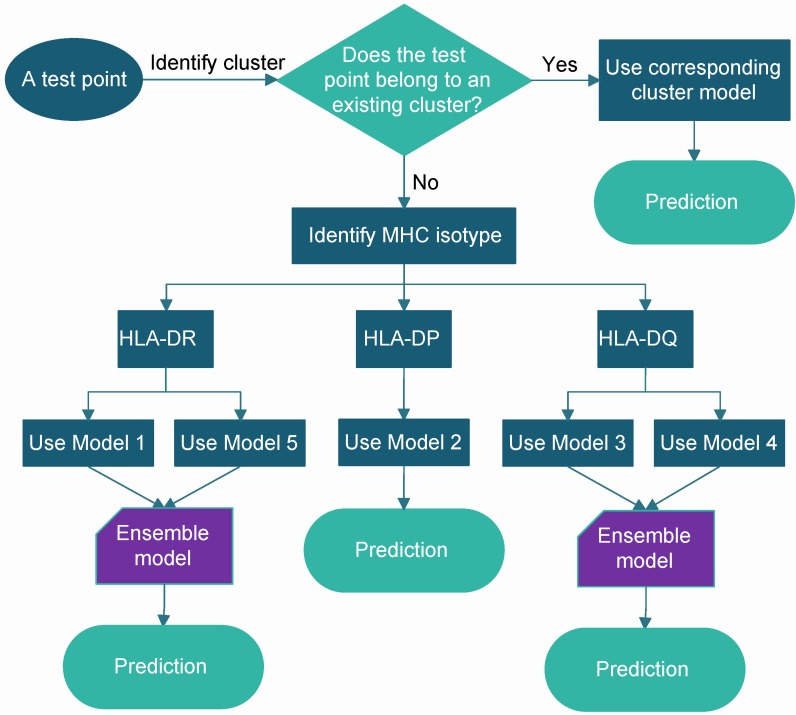
Schematic illustration of making a prediction on a test point.

In the following section, we present the results of a novel ensemble method, called superMHC, for class II MHC-peptide binding prediction, including three class II HLA isotypes of HLA-DR, HLA-DP, and HLA-DQ. The NetMHCIIpan-3.2 dataset was used for evaluation. The superMHC method is a pan-allele approach, which can make accurate predictions of those class II HLA molecules with few binders available by making use of those MHC-peptide pairs having experimental measurements. A schematic overview of the superMHC model construction is shown in Figure 4. Basically, the model construction was composed of two steps. First, we classified the training set into five clusters. In Section 2.1, four main supertypes are identified for molecules from the HLA-DR, HLA-DP, and HLA-DQ loci. Based on the broad supertype classification, all the samples in the training set were partitioned into five parts by mapping their MHC molecules to the main supertypes, in which four parts were associated with the corresponding four main supertypes and the remaining samples were associated with a single cluster, named diverse cluster, molecules that have no broad supertype classification herein. Second, a separate regularized least squares (RLS) regression [21] model was then trained on each of these five clusters. After five base learners were produced, making a prediction of a point from the test set was as illustrated in Figure 5, which involved two steps:

1. If the MHC molecule of the test point belongs to one of the five existing clusters, then use the corresponding cluster model to perform prediction for the test point.
2. Otherwise, identify the MHC isotype of the test point. The base learners Model 1 and Model 5 associated with the main DR and diverse clusters respectively were combined to make a prediction for the test point from the HLA-DR isotype. Since about 66% of data points associated with the diverse cluster in the training set were from the HLA-DR isotype, Model 1 was combined with Model 5 to perform the prediction. The base learner Model 2 associated with the main DP cluster was applied to perform prediction for the test point from HLA-DP isotype. The base learners Model 3 and Model 4 associated with the main DQ1 and main DQ2 clusters, respectively, were combined to perform prediction for the test point from the HLA-DQ isotype.

We validated superMHC on the NetMHCIIpan-3.2 dataset through five-fold cross-validation. The NetMHCIIpan-3.2 dataset was partitioned into the same five folds as [11]. In each test, we merged four parts of the data objects as the training set and left the other part as the test set. The pan-allele kernel ${\hat{K}}_{\mathrm{PAN}}^{3}$ in Equation (8) was defined by using $\beta_{peptide}^{*}=0.1137$ and $\beta_{allele-b}^{*}=0.06$, as suggested in [18], to define ${\hat{K}}_{P}^{3}\left(p,p^{\prime }\right)$ and ${\hat{K}}_{B}^{3}\left(b,b^{\prime }\right)$, while $\beta_{allele-a}$ for ${\hat{K}}_{A}^{3}\left(a,a^{\prime }\right)$ and λ for RLS were chosen from sets $\left\{0.02\times n:n=1,2,3,4\right\}⋃\left\{e^{n}:n=-15,-14,\cdots ,-11\right\}$. We found that the model performed best with $\beta_{allele-a}^{*}=0.02$ and $\lambda^{*}=e^{-13}$. The predictive performances of superMHC compared with the NetMHCIIpan-3.2, NetMHCII-2.3, and their consensus method are shown in Table 3. The performance of superMHC was significantly better than NetMHCII-2.3 (p<0.05, paired *t*-test). The average AUC scores over all 54 MHC molecules were 0.840 and 0.857 for NetMHCII-2.3 and superMHC, respectively. The performance of superMHC was comparable to those of NetMHCIIpan-3.2 and the consensus method.

To investigate the generalization ability of the superMHC method, we used the whole NetMHCIIpan-3.2 dataset for training and tested its predictive performance on a new dataset. To avoid overlapping between the training and testing sets, those MHC-peptide pairs overlapping with the training set were removed. The performance of superMHC in comparison with the NetMHCII-2.3, NetMHCIIpan-3.2, and their consensus method is given in Table 4. Since some molecules in the test set were insufficient to define the AUC scores, we compared the algorithm performance in terms of RMSE, which is a better measure than AUC, as suggested in [18]. The NetMHCII-2.3 and consensus method could only make the prediction in 9 out of 33 MHC molecules. For these 9 molecules, the performance of superMHC in comparison with the other three methods was comparable. Both superMHC and NetMHCIIpan-3.2 were able to perform prediction for all 33 molecules. Comparing the performance of superMHC with NetMHCIIpan-3.2, we found that their RMSE scores were not significantly different (p>0.05, paired *t*-test). Specifically, among the four predicted methods, superMHC achieved the best performance for 15 molecules and NetMHCIIpan-3.2 performed the best for 14 molecules. The smallest RMSE score for each molecule is highlighted in bold in Table 4.

## 3. Materials and Methods

### 3.1. Datasets

#### 3.1.1. MHC II-Peptide Binding Repertoires

A large-scale peptide-binding dataset containing 72 human class II MHC molecules was considered in this study. This dataset was obtained from the NetMHCIIpan-3.2 web server [11], and all mouse H-2 molecules were excluded. The NetMHCIIpan-3.2 dataset covers 131,008 MHC-peptide pairs, in which there are 36 HLA-DR, 27 HLA-DQ, and 9 HLA-DP molecules, which were used as the training set in this study.

A new test set of class II HLA-peptide binding data was downloaded from the Immune Epitope Database (IEDB) [22] to verify the performance of the superMHC method. We retrieved all quantitative data by including either radioactivity or fluorescence competition binding assays with half maximal inhibitory concentration (IC${}_{50}$) response. To avoid overlapping between the training and testing sets, those MHC-peptide pairs overlapping with the training set were removed. Since both the NetMHCII-2.3 and NetMHCIIpan-3.2 methods cannot perform the prediction for peptides of less than 9 amino acids, those MHC-peptide pairs with a peptide length less than 9 were excluded as well. In addition, we just included those MHC molecules with more than five measured peptides. Finally, the new test set was composed of 33 MHC molecules and 8892 pairs covering the HLA-DR, HLA-DP, and HLA-DQ loci. Most publicly-available peptide-binding data from the HLA-DP and HLA-DQ loci have been included in the training set; hence, the new test set covers limited data from these two loci. The IC${}_{50}$ scores usually lie between zero and 50,000 nanomolar (nM), which measured the binding strength of a peptide binding to an MHC molecule, and are normalized by Equation (1).

(1) $$\psi \left(IC_{50}\right)=\left\{\begin{array}{cc} 0 & IC_{50}>50,000,\\ 1-\log_{50,000}IC_{50} & 1\leq IC_{50}\leq 50,000,\\ 1 & IC_{50}<1. \end{array}\right.$$

#### 3.1.2. MHC II Sequences

The aligned protein sequences of the class II MHC molecules were downloaded from the IMGT/HLA Sequence Database. Two markers listed in Table 5 were used to identify the polymorphic part of a class II MHC allele, each of which consists of three amino acids. For each allele, we only consider the amino acids located from the “start marker” to the “end marker” since this region constitutes the whole of exon 2. The class II MHC gene exon 2 encodes the peptide-binding sites, thereby contributing to the diversity in antigen presentation [23,24,25]. The DRA (HLA-DR α-chain) allele is very monomorphic; in contrast, both the DQA (HLA-DQ α-chain) and DPA (HLA-DP α-chain) alleles contain the polymorphisms specifying the peptide binding specificities [26], so we therefore considered the polymorphisms of both the α and β chains in the superMHC model development.

### 3.2. Methods

#### 3.2.1. Analysis of Peptide-Binding Repertoire Dissimilarity

In this study, the large-scale dataset studied in [11] was utilized to generate the peptide-binding repertoires. The peptide-binding dataset contains 41 class II HLA molecules and 96,674 MHC-peptide pairs, as shown in Table 1. All MHC-peptide pairs in the dataset have an IC${}_{50}$ score between 1 nM and 50,000 nM. Furthermore, each MHC molecule in the dataset has at least 200 peptides with known binding affinities. We developed the RDI as a measure of distance between repertoires to quantify the difference in MHC binding specificities. There are a number of challenges associated with the quantification of overlapping peptide-binding repertoires. First, the high variance in different MHC peptide-binding repertoires can often result in orders of magnitude difference in counts of sharing peptides. In addition, MHC molecules with small binding repertoires display very limited overlap with other molecules. To account for these challenges and to make meaningful comparisons between repertoires, the RDI was defined in three steps.

1. Let P and M be finite sets of peptides and MHCs, respectively. Suppose $\bar{Z}={\left\{\left(P_{i},F_{i}\right)\right\}}_{i=1}^{m}$ is a sample set of peptide-binding repertoires with $P_{i}\subset P$ and $F_{i}\subset R$; for each MHC molecule $M_{i}$, $f_{pi}$ represents the normalized binding affinity (see Equation (1)) between peptide *p* and MHC molecule $M_{i}$. Here, the number of peptides shared by MHC molecules $M_{i}$ and $M_{j}$ is denoted by $|P_{ij}|$, where $P_{ij}=P_{i}\cap P_{j}$.
2. Calculate the average absolute difference between the normalized binding affinities given by the two MHC molecules $M_{i}$, $M_{j}$ and their shared peptides. The difference is defined as:
  (2) $$d_{ij}=\frac{1}{|P_{ij}|}\sum_{p\in P_{ij}}\left|f_{pi}-f_{pj}\right|$$
3. Subsequently, we defined the RDI to quantify the dissimilarity in binding specificity by transforming Kendall’s rank correlation coefficient, which is more robust to outliers compared to Pearson correlation [27]. This metric employs Kendall’s rank correlation coefficient to evaluate the degree of similarity between two sets of ranks given to the same set of objects. The value of the correlation coefficient varies between −1 and 1.
  (3) $$r_{K}=\frac{2}{\left|M|(|M|-1\right)}\sum_{\begin{array}{c} k<k^{\prime }\\ M_{k},M_{k^{\prime }}\in M \end{array}}sign\left(\left(d_{ki}-d_{k^{\prime }i}\right)\left(d_{kj}-d_{k^{\prime }j}\right)\right),\forall M_{i},M_{j}\in M.$$
  where $\left\{\left(d_{ui},d_{uj}\right),M_{u}\in M\right\}$ is a set of pairs of differences between MHC molecules $M_{u}$ and $M_{i},M_{j}$, as defined in Equation (2). This coefficient depends on only the order of the pairs.
  The RDI is given by:
  (4) $$RDI=1-r_{K},\forall M_{i},M_{j}\in M.$$

#### 3.2.2. Identification of Supertypes for MHC II Molecules

Class II HLA supertypes were obtained by clustering the peptide-binding repertoires. First, the RDI was utilized to measure the difference in peptide-binding specificities for all distinct pairs of class II HLA molecules. WPGMA (weighted pair-group method using arithmetic averages) linkage was used to measure the proximity between clusters. Suppose $P^{\prime }$ and $P^{\prime \prime }$ are merged into a new cluster *P*, then the proximity between *P* and another cluster *Q* is defined as follows:

(5) $$\Delta \left(P,Q\right)=\frac{\Delta \left(P^{\prime },Q\right)+\Delta \left(P^{\prime \prime },Q\right)}{2}$$

Then, hierarchical agglomerative clustering was applied to build a cluster tree, which is a tree on which every node represents the cluster of the set of all leaves descending from that node, and the relationship was visualized in the form of a cluster tree. Finally, class II HLA supertypes were identified by cutting the cluster tree at a proper height to classify the molecules into disjoint subsets.

#### 3.2.3. Ensemble Learning

Following from the previous section, clustering the MHC molecules gives a compressed representation, the MHC molecules assigned in the same supertype displaying higher functional similarity, while the MHC molecules in different supertypes displaying very limited functional overlap. This transformation tells us something interesting about the structure of the data, and in this study, we exploited it to improve the predictive performance of class II MHC-peptide binding. We trained a separate predictor on each cluster rather than training a single predictor on the entire dataset. The separate predictors were then properly combined to generate an ensemble predictor. We regard ensemble learning as sets of machine learning approaches whose decisions are integrated in a proper way to enhance the whole system’s performance [28,29,30]. The generalization ability of an ensemble is usually much better than that of a single learner [31,32].

The construction of an ensemble predictor comprises the following three steps:

1. The whole training set of peptide-binding repertoires was clustered into *K* (K=5 herein) disjoint subsets.
2. For each subset, the RLS supervised learning algorithm was trained on the data objects inside it. We then obtained a set of *K* separate predictors.
3. An ensemble predictor was generated by combining the separate predictors derived from the same MHC isotype by uniform averaging.

#### 3.2.4. Pan-Allele Kernel and RLS Regression

According to the definition of a $K^{3}$ string kernel in [18], it can be used as a measure of similarity between amino acid sequences, and two sequences are considered similar if they contain many high-score local alignments. We briefly review the kernel definition herein. Given two amino acid sequences *f* and *g*, the string kernel $K^{3}$ is defined as:

(6) $$K^{3}\left(f,g\right)=\sum_{\begin{array}{c} u\subset f,v\subset g\\ |u|=|v|=k\\ \mathrm{all}\;k=1,2,\ldots \end{array}}\prod_{i=1}^{k}{\left(\frac{Q(u_{i},v_{i})}{p\left(u_{i}\right)p\left(v_{i}\right)}\right)}^{\beta },\;forsome\beta >0$$

where Q(x,y) represents the frequency of a *x* to *y* amino acids substitution in the alignment blocks to generate a BLOSUM62 substitution matrix [33], $p\left(x\right)=\sum_{y\in A}Q\left(x,y\right)$, A is a set of 20 amino acids, and *u* and *v* are substrings of *f* and *g*, respectively, of the same length *k*.

With correlation normalization:

(7) $$\begin{array}{c} {\hat{K}}^{3}\left(f,g\right)=\frac{K^{3}\left(f,g\right)}{\sqrt{K^{3}\left(f,f\right)K^{3}\left(g,g\right)}}. \end{array}$$

Each HLA class II molecule consists of two chains of α and β. For HLA-DR, the β chain is highly polymorphic, while the α chain is closely monomorphic. Different from HLA-DR, both HLA-DP and HLA-DQ contain the polymorphism in α and β chains, which specify the peptide binding specificities. Therefore, for HLA-DP and HLA-DQ, both α and β chains should be taken into account to predict peptide binding. The pan-allele kernel proposed in [18] solely considered the β chain of the MHC molecules. In this study, this pan-allele kernel was further explored to take both α and β chains into account. For all HLA-DR molecules, the same α chain of DRA*01:01 was adopted.

Let A and B be finite sets of amino acid sequences representing the MHC II α and β chains, respectively. Let P be a set of peptides. We define the pan-allele kernel on the product space of A×B×P as:

(8) $${\hat{K}}_{\mathrm{PAN}}^{3}\left(\left(a,b,p\right),\left(a^{\prime },b^{\prime },p^{\prime }\right)\right)={\hat{K}}_{A}^{3}\left(a,a^{\prime }\right){\hat{K}}_{B}^{3}\left(b,b^{\prime }\right){\hat{K}}_{P}^{3}\left(p,p^{\prime }\right).$$

The kernel ${\hat{K}}_{\mathrm{PAN}}^{3}$ was implemented with RLS for MHC-peptide binding prediction. Let a set of data ${\left\{\left(x_{i},y_{i}\right)\right\}}_{i=1}^{m}$ be given, where for each *i*, $x_{i}=\left(a_{i},b_{i},p_{i}\right)$ with $a_{i}\in A,b_{i}\in B,p_{i}\in P$, and $y_{i}\in \left[0,1\right]$ is the normalized binding affinity of peptide $p_{i}$ to class II MHC molecule $\left(a_{i},b_{i}\right)$. The problem comes to solve:

(9) $$\bar{f}=\arg\min_{f\in H_{K}}\sum_{i=1}^{m}{\left(f\left(x_{i}\right)-y_{i}\right)}^{2}+\lambda {∥f∥}_{K}^{2},\;for\lambda >0.$$

where $K={\hat{K}}_{\mathrm{PAN}}^{3}$, and $f(x_{i})$ is the predicted binding affinity of peptide $p_{i}$ to class II MHC molecule $\left(a_{i},b_{i}\right)$.

The prediction at a data point $x^{*}$ will be given by [21]:

(10) $$f\left(x^{*}\right)=\sum_{i=1}^{m}c_{i}K\left(x_{i},x^{*}\right),\;\mathrm{for}\;\mathrm{some}c_{i}\in R$$

#### 3.2.5. Performance Measures

Predictive performance of MHC II peptide binding was evaluated using the root mean squared error (RMSE), as well as the area under the receiver operating characteristics curve (AUC). A smaller RMSE or higher AUC score reflects a better performance. We used a binding threshold of 500 nM to evaluate the AUC score, which is between 0 and 1, where the AUC score is equal to 1 for a perfect classifier and 0.5 for a random classifier.

A paired *t*-test was used for statistical comparison, and the score comparison result is considered to be statistically significant if *p* is less than 0.05.

## 4. Conclusions

The T lymphocytes are one type of the central cells of adaptive immunity, typically of cell-mediated immunity. T cell receptors only recognize antigenic peptides that are bound to MHC molecules, thus peptides displayed by MHC molecules comprise a pivotal process to activate T cells. The binding measurement by chemical and biological experiments is time consuming and expensive; hence, many computational tools have been developed for this binding prediction. In the present study, we have developed a new method, called superMHC, for class II MHC-peptide binding prediction by using supertype clustering in conjunction with RLS regression. By using the kernel-based RLS, we need to create the kernel matrix *K* by computing all pairwise similarities, which is memory intensive and speed consuming for very large datasets. The conjunction of RLS regression with supertype clustering enables building individual RLS regression models on a much smaller subset of the data, thus reducing the memory and speed usage.

We utilized a large-scale dataset derived from quantitative MHC binding assays to identify clusters, or supertypes, from the 41 most common class II human MHC molecules covering the DR, DP, and DQ loci. The dissimilarity in the binding specificity of any two MHC molecules was quantified by a novel RDI based on Kendall’s rank correlation coefficient. Our results identified two main supertypes for HLA-DQ and one each for HLA-DR and HLA-DP. However, we did not include all class II MHC molecules in the cluster tree construction; therefore, more supertypes might be identified in the future when more MHC molecules are considered. The identification of supertypes provides a compressed representation, and the MHC molecules assigned in different main supertypes display very limited binding repertoire overlap or functional overlap. The supertype clustering was done in a completely unsupervised way without any regard to the target. These four main supertypes and a diverse cluster have been employed to derive five base learners. The superMHC method is a more complex model that contains five base learners herein. An important issue about the ensemble method was choosing which predictions to average. The methodology chosen in this study was a uniform averaging of the predictions made by the base learners derived from the same MHC isotype. The choice of combining the predictors derived from the same MHC isotype was due to the observation that class II MHC molecules from different loci display very limited binding repertoire overlap.

There are very limited methods available for pan-allele HLA-DR, HLA-DP, and HLA-DQ binding prediction. It is more difficult to develop a cross-loci method for class II MHC molecules due to the differences of the polymorphisms and binding motifs of different loci. The superMHC method is a pan-allele method that is able to make accurate prediction for the three isotypes of HLA-DR, HLA-DP, and HLA-DQ. Both HLA-DP and HLA-DQ molecules contain the polymorphism in α and β chains that contributes to the diversity in antigen presentation; thus, the superMHC method considers both the α and β chains in the model construction by defining a pan-allele kernel on the product space of MHC α chains, β chains, and peptides. In addition, only a few MHC molecules have sufficient measured peptides for construction of a reliable prediction model up to date, and the pan-allele kernel enabled us to make an accurate prediction of those MHC molecules with few binders available. We compared the superMHC method with the state-of-the-art NetMHCII-2.3 and NetMHCIIpan-3.2 methods, both of which have been shown to be among the best methods for MHC II binding prediction [11,34]. Both the NetMHCII-2.3 and NetMHCIIpan-3.2 methods are based on artificial neural networks. The NetMHCII-2.3 method is a fixed-allele method that can only make predictions of 25 HLA-DR, 9 HLA-DP, and 20 HLA-DQ molecules. The same as the superMHC method, the NetMHCIIpan-3.2 method is a pan-allele method, which integrates information of both peptides and MHC molecules and is capable of predicting binding affinities to all class II HLA molecules with a known primary sequence. The NetMHCIIpan-3.2 method considered information of class II MHC molecules using a binding pocket pseudo-sequence of 34 residues in length; however, the superMHC method incorporated the continuous region covering the whole of exon 2, which encodes the peptide-binding sites. We first evaluated superMHC on the NetMHCIIpan-3.2 dataset by five-fold cross-validation, and the results show that the performance of superMHC is significantly better than NetMHCII-2.3, while comparable with NetMHCIIpan-3.2 and the consensus method of NetMHCII-2.3 and NetMHCIIpan-3.2. Next, we used the whole NetMHCIIpan-3.2 dataset for training and validated superMHC on a new test set downloaded from the Immune Epitope Database (IEDB). This test set has no MHC-peptide pairs overlapping with the training set. The NetMHCII-2.3 and the consensus method of NetMHCII-2.3 and NetMHCIIpan-3.2 can only make the prediction in 9 out of 33 MHC molecules in the test set, while superMHC and NetMHCIIpan-3.2 can make the prediction for the whole test set covering three MHC II isotypes. The performance of superMHC in comparison to NetMHCIIpan-3.2 is not significantly different in terms of RMSE (*P* > 0.05, paired *t*-test). Specifically, the superMHC method achieves the best performance in 15 out of 33 MHC molecules among the four compared methods.

In summary, the purpose of this work was to present a framework of the utility of supertype clustering to gain more information about the data to improve the prediction accuracy of class II MHC-peptide binding. The results show that the ensemble method superMHC achieves the state-of-the-art performance. This ensemble learning framework of combining supertype clustering with RLS regression is applicable to other base learning algorithms, which can be support vector machines, neural networks, or other kinds of machine learning algorithms.

## Figures and Tables

**Table 1:** Overview of the class II MHC peptide-binding repertoires.

Allele	#Peptides	#Binders
DRB1_0101	9605	6049
DRB1_0401	5834	2984
DRB1_0404	3361	1846
DRB1_0701	5857	3418
DRB1_0802	4160	2023
DRB1_0901	4090	2146
DRB1_1001	1995	1485
DRB1_1101	5320	2646
DRB1_1302	4061	2178
DRB1_1501	4374	2051
DRB1_1602	1643	977
DRB3_0301	778	504
DRB4_0101	3634	1520
DRB4_0103	700	522
DRB5_0101	4495	2381
HLA-DPA10103-DPB10201	674	139
HLA-DPA10103-DPB10301	1280	566
HLA-DPA10103-DPB10401	2251	637
HLA-DPA10103-DPB10601	437	240
HLA-DPA10201-DPB10101	2272	808
HLA-DPA10201-DPB10501	2119	710
HLA-DPA10201-DPB11401	2017	844
HLA-DPA10301-DPB10402	2381	879
HLA-DQA10101-DQB10501	2489	815
HLA-DQA10102-DQB10502	643	156
HLA-DQA10102-DQB10602	2599	1253
HLA-DQA10103-DQB10603	255	90
HLA-DQA10104-DQB10503	723	105
HLA-DQA10201-DQB10202	793	119
HLA-DQA10201-DQB10301	624	374
HLA-DQA10201-DQB10303	554	265
HLA-DQA10201-DQB10402	570	238
HLA-DQA10301-DQB10302	2812	568
HLA-DQA10303-DQB10402	323	117
HLA-DQA10401-DQB10402	2707	928
HLA-DQA10501-DQB10201	2663	873
HLA-DQA10501-DQB10301	3474	1809
HLA-DQA10501-DQB10302	680	203
HLA-DQA10501-DQB10303	461	179
HLA-DQA10501-DQB10402	614	336
HLA-DQA10601-DQB10402	352	132
Total	96,674	46,113

**Table 2:** Broad supertypes, split supertypes, and binding motifs.

Broad Supertype	Split Supertype	HLA Molecule	Common Binding Motifs								
			p1	p2	p3	p4	p5	p6	p7	p8	p9
		DRB3*0301									
	DR52	DRB1*1302	ILVF			NS		ASN			VIL
		DRB1*0101									
		DRB1*1001									
		DRB1*0701									
		DRB1*0901									
	DR1	DRB1*1602	FYIL			LAIST		ASGPT			LV
		DRB1*0401									
		DRB1*0404									
main DR		DRB1*1501									
		DRB1*0802									
		DRB5*0101									
	DR53	DRB1*1101									
		DRB4*0101	FILY	R		LAIV	R	AR	RL	R	R
		DRB4*0103									
		HLA-DQA10501-DQB10301									
		HLA-DQA10201-DQB10301									
		HLA-DQA10102-DQB10602									
		HLA-DQA10103-DQB10603									
	DQ7	HLA-DQA10201-DQB10303	AVI		AGTS	AS		AVPI			ASVG
		HLA-DQA10501-DQB10302									
		HLA-DQA10501-DQB10303									
		HLA-DQA10201-DQB10402									
main DQ1		HLA-DQA10303-DQB10402									
	DQ4	HLA-DQA10601-DQB10402	FLIV			FAW		AVPIT	RA		ASLV
		HLA-DQA10501-DQB10402									
		HLA-DQA10301-DQB10302									
	DQ8	HLA-DQA10401-DQB10402				AVI		AVI	EAL	EAD	ED
		HLA-DQA10102-DQB10502									
	DQ5	HLA-DQA10104-DQB10503	LIF			FYW	F	DLIV	D		
main DQ2		HLA-DQA10101-DQB10501									
		HLA-DQA10501-DQB10201									
	DQ2	HLA-DQA10201-DQB10202	FLIV			AVILD		AVID	ED	ED	ELD
		HLA-DPA10103-DPB10401									
		HLA-DPA10201-DPB10101									
		HLA-DPA10201-DPB10501									
main DP	DP1	HLA-DPA10301-DPB10402	FLYI					FLYI	LF		L
		HLA-DPA10103-DPB10201									
		HLA-DPA10103-DPB10601									
		HLA-DPA10103-DPB10301									
	DP3	HLA-DPA10201-DPB11401	FLRIY	RK		ALS		APVSI	AL		

**Table 3:** The performance of superMHC in comparison with NetMHCII-2.3, NetMHCIIpan-3.2, and their consensus method on the NetMHCIIpan-3.2 dataset in terms of AUC.

**MHC Molecules**	**# of Peptides**	**NetMHCII-2.3**	**NetMHCIIpan-3.2**	**Consensus (Net)**	**superMHC (AUC)**	**superMHC (RMSE)**
DRB1_0101	10412	0.829	0.832	0.838	0.835	0.197
DRB1_0103	42	0.250	0.678	0.599	0.697	0.178
DRB1_0301	5352	0.816	0.816	0.826	0.831	0.183
DRB1_0401	6317	0.798	0.809	0.813	0.809	0.199
DRB1_0402	53	0.633	0.701	0.649	0.663	0.229
DRB1_0403	59	0.644	0.841	0.787	0.830	0.140
DRB1_0404	3657	0.787	0.812	0.808	0.811	0.189
DRB1_0405	3962	0.839	0.827	0.846	0.836	0.173
DRB1_0701	6325	0.877	0.875	0.885	0.880	0.173
DRB1_0801	937	0.834	0.844	0.854	0.834	0.170
DRB1_0802	4465	0.834	0.834	0.844	0.842	0.182
DRB1_0901	4318	0.832	0.833	0.843	0.831	0.175
DRB1_1001	2066	0.912	0.923	0.924	0.910	0.157
DRB1_1101	6045	0.867	0.864	0.873	0.865	0.175
DRB1_1201	2384	0.891	0.868	0.892	0.883	0.146
DRB1_1301	1034	0.828	0.857	0.856	0.855	0.225
DRB1_1302	4477	0.889	0.885	0.895	0.883	0.184
DRB1_1501	4850	0.833	0.834	0.842	0.842	0.188
DRB1_1602	1699	0.879	0.883	0.888	0.883	0.154
DRB3_0101	4633	0.898	0.888	0.900	0.891	0.160
DRB3_0202	3334	0.887	0.869	0.886	0.873	0.183
DRB3_0301	884	0.824	0.840	0.845	0.818	0.198
DRB4_0101	3961	0.837	0.822	0.844	0.852	0.171
DRB4_0103	846	0.839	0.841	0.861	0.867	0.193
DRB5_0101	5125	0.849	0.849	0.858	0.854	0.192
HLA-DPA10103-DPB10201	787	0.910	0.917	0.921	0.920	0.144
HLA-DPA10103-DPB10301	1563	0.914	0.902	0.916	0.914	0.165
HLA-DPA10103-DPB10401	2725	0.935	0.935	0.939	0.936	0.144
HLA-DPA10103-DPB10402	45	0.497	0.710	0.636	0.515	0.186
HLA-DPA10103-DPB10601	584	0.996	0.995	0.995	0.996	0.105
HLA-DPA10201-DPB10101	2447	0.903	0.903	0.909	0.899	0.145
HLA-DPA10201-DPB10501	2470	0.914	0.911	0.919	0.915	0.153
HLA-DPA10201-DPB11401	2302	0.937	0.930	0.938	0.940	0.151
HLA-DPA10301-DPB10402	2641	0.906	0.904	0.910	0.900	0.158
HLA-DQA10101-DQB10501	2946	0.917	0.900	0.917	0.916	0.144
HLA-DQA10102-DQB10501	833	0.867	0.839	0.874	0.869	0.192
HLA-DQA10102-DQB10502	800	0.850	0.835	0.859	0.868	0.157
HLA-DQA10102-DQB10602	2747	0.905	0.890	0.906	0.893	0.152
HLA-DQA10103-DQB10603	462	0.816	0.861	0.855	0.856	0.186
HLA-DQA10104-DQB10503	883	0.844	0.805	0.844	0.840	0.145
HLA-DQA10201-DQB10202	944	0.851	0.814	0.853	0.838	0.133
HLA-DQA10201-DQB10301	827	0.864	0.849	0.871	0.857	0.195
HLA-DQA10201-DQB10303	761	0.887	0.894	0.899	0.891	0.150
HLA-DQA10201-DQB10402	768	0.858	0.860	0.875	0.857	0.186
HLA-DQA10301-DQB10301	207	0.761	0.839	0.814	0.875	0.183
HLA-DQA10301-DQB10302	3111	0.849	0.810	0.842	0.854	0.125
HLA-DQA10303-DQB10402	567	0.836	0.820	0.855	0.835	0.176
HLA-DQA10401-DQB10402	2890	0.894	0.883	0.897	0.894	0.117
HLA-DQA10501-DQB10201	2897	0.889	0.876	0.888	0.882	0.133
HLA-DQA10501-DQB10301	3585	0.922	0.915	0.924	0.922	0.143
HLA-DQA10501-DQB10302	847	0.831	0.822	0.840	0.824	0.136
HLA-DQA10501-DQB10303	564	0.884	0.876	0.892	0.887	0.132
HLA-DQA10501-DQB10402	749	0.857	0.868	0.876	0.863	0.166
HLA-DQA10601-DQB10402	565	0.845	0.848	0.872	0.859	0.187
Average		0.840	0.854	0.861	0.857	0.167

Consensus (Net) represents the consensus method by averaging the prediction scores from NetMHCII-2.3 and NetMHCIIpan-3.2. The performance of superMHC in terms of RMSE is given in the last column.

**Table 4:** Predictive performance of the superMHC method compared with those of NetMHCII-2.3, NetMHCIIpan-3.2, and their consensus method on the new test set in terms of RMSE.

**MHC Molecules**	**#Peptides**	**NetMHCII-2.3**	**NetMHCIIpan-3.2**	**Consensus (Net)**	**superMHC**
DRB1_0101	1427	0.241	0.244	**0.240**	0.249
DRB1_0301	912	0.235	**0.222**	0.224	0.230
DRB1_0302	148		0.260		**0.233**
DRB1_0401	1392	**0.210**	0.221	0.212	0.238
DRB1_0402	6	0.351	**0.238**	0.288	0.292
DRB1_0404	34	0.211	0.203	0.203	**0.178**
DRB1_0405	14	**0.202**	0.236	0.213	0.229
DRB1_0701	125	0.284	**0.277**	0.277	0.278
DRB1_0801	9	0.186	0.126	0.153	**0.105**
DRB1_0806	118		0.363		**0.326**
DRB1_0813	1370		0.251		**0.229**
DRB1_0819	116		0.213		**0.206**
DRB1_0901	28	**0.200**	0.256	0.223	0.260
DRB1_1101	163		**0.338**		0.350
DRB1_1104	7		0.339		**0.292**
DRB1_1201	115		**0.335**		0.340
DRB1_1202	124		0.370		**0.353**
DRB1_1301	9		**0.352**		0.399
DRB1_1302	17		**0.371**		0.423
DRB1_1402	125		0.255		**0.237**
DRB1_1404	30		0.198		**0.193**
DRB1_1412	116		**0.310**		0.313
DRB1_1501	132		**0.298**		0.300
DRB1_1502	6		**0.361**		0.447
DRB1_1601	16		0.240		**0.233**
DRB3_0101	41		0.281		**0.242**
DRB3_0301	159		**0.297**		0.313
DRB4_0101	18		**0.193**		0.207
DRB5_0101	1331		**0.226**		0.237
DRB5_0102	8		0.231		**0.219**
DRB5_0202	16		**0.190**		0.233
HLA-DPA10103-DPB10201	751		0.126		**0.118**
HLA-DQA10302-DQB10301	9		0.203		**0.045**
Average I	8892	0.236	0.225	0.226	0.229
Average II			0.261		0.259

“Average I” is calculated over 9 MHC molecules covered by NetMHCII-2.3. “Average II” is calculated over all 33 MHC molecules. The smallest RMSE score in each row is marked in bold.

**Table 5:** Start and end markers to identify the polymorphic region of an allele.

	**Alpha Chain**			**Beta Chain**		
Loci	DPA	DQA	DRA	DPB	DQB	DRB
Start	DHV	DHV	EHV	NYL/NYV	DFV	RFL
End	AAN/ATN	ATN	ITN	QRR	QRR	QRR

“Start marker” represents the location of its first occurrence in the allele. “End marker” represents the location of its last occurrence in the allele.
